# Original and Modified Sauvé–Kapandji Procedures: A Two-Axis Framework for Surgical Decision-Making—A Narrative Review

**DOI:** 10.3390/jcm15166368

**Published:** 2026-08-18

**Authors:** Il-Jung Park, Youn-Tae Roh

**Affiliations:** 1 Department of Orthopaedic Surgery, Bucheon St. Mary’s Hospital, College of Medicine, The Catholic University of Korea, Seoul 06591, Republic of Korea; jikocmc@catholic.ac.kr; 2 Department of Orthopaedic Surgery, Seoul St. Mary’s Hospital, College of Medicine, The Catholic University of Korea, Seoul 06591, Republic of Korea

**Keywords:** Sauvé–Kapandji procedure, distal radioulnar joint, rheumatoid arthritis, ulnar stump instability, surgical technique, decision-making framework, narrative review

## Abstract

The Sauvé–Kapandji (SK) procedure is a salvage operation that fuses the distal radioulnar joint (DRUJ) and creates an intentional pseudarthrosis in the proximal ulna, thereby relieving pain while preserving forearm rotation and ulnar-sided bony support. This narrative review (1) traces the historical evolution of the original and modified SK procedures, (2) organizes the modifications within a two-axis framework—distal bone handling and proximal soft-tissue stabilization—to clarify their indications and outcomes, and (3) proposes an indication-based decision framework keyed to bone quality and etiology, together with a research agenda to validate it. Along the bony axis, the technique differentiates stepwise according to bone quality—traditional SK, the fragment-interposition (Nakamura) type, and the ulnar-head rotation (Fujita) type; along the soft-tissue axis, the amount and position of ulnar resection appear to be the more fundamental determinants of stump stability. SK and the Darrach procedure are broadly equivalent in major outcomes, but SK may be advantageous in patients at risk of ulnar carpal translation or with high functional demand. Given that the evidence is predominantly Level IV, this two-axis framework is offered not as an established guideline but as a hypothesis-generating conceptual framework requiring validation by future comparative research. As a narrative review, this work was not designed as a systematic review or meta-analysis and does not include a registered protocol, formal quality appraisal, or quantitative data pooling.

## 1. Introduction

Dysfunction of the distal radioulnar joint (DRUJ) is a common and not infrequently disabling source of ulnar-sided wrist pain, weakness, and limited forearm rotation, arising from a broad range of conditions including rheumatoid arthritis (RA), primary and post-traumatic osteoarthritis, instability after distal radius malunion, and congenital deformities such as Madelung deformity [1,2]. When conservative treatment fails, a salvage procedure is considered by balancing the patient’s functional demand against the extent of bony resection; the options range from hemiresection–interposition arthroplasty to complete distal ulnar resection (Darrach), the Sauvé–Kapandji (SK) procedure, and joint arthroplasty [2].

Among these, the SK procedure fuses the DRUJ while creating a pseudarthrosis in the proximal ulna, eliminating pain yet preserving forearm rotation, and—unlike the Darrach procedure, which resects the entire distal ulna—it preserves the ulnar head and its supporting structures [3]. For this reason, the SK procedure has been favored in young, active patients and in rheumatoid wrists at risk of ulnar carpal translation [4,5]. The original SK procedure, however, can be complicated by symptomatic instability of the proximal ulnar stump, which has necessitated revision in up to 18% of patients in one comparative series and in 6 of 15 wrists in a 13-year series [6,7], as well as by nonunion of the arthrodesis [8]. To address these problems, numerous bony and soft-tissue modifications have been introduced; yet no study has compared them head-to-head or integrated them into a structured decision framework, and even recent higher-level syntheses compare only SK with the Darrach procedure [9,10,11]. As a result, the choice among modifications still relies on conventional judgments of age and demand rather than on consistent evidence [6].

This review therefore aims to (1) trace the historical evolution of the original and modified SK procedures, (2) organize the modifications within a two-axis framework—distal bone handling and proximal soft-tissue stabilization—to define the indications and outcomes of each, and (3) propose a clinically oriented, indication-based decision framework together with a research agenda for its validation.

## 2. Materials and Methods

This work is a narrative review. Given the marked heterogeneity of the available literature, in underlying etiology, technical variant, fixation method, and outcome measures, and the predominance of small, retrospective (Level IV) series, a narrative review was considered more appropriate than a systematic review or meta-analysis for developing a clinically oriented conceptual framework; the primary objective was to integrate the existing modifications into such a framework rather than to derive pooled estimates of treatment effect. Accordingly, no protocol was registered, and no formal quality appraisal or quantitative data pooling was performed.

The English-language literature on the Sauvé–Kapandji procedure was searched in PubMed/MEDLINE and Embase, supplemented by Google Scholar and by hand-searching the reference lists of relevant articles, covering records from database inception through 16 June 2026, which was the date of the final literature search. Only English-language publications were considered. Search terms combined “Sauvé–Kapandji,” “distal radioulnar joint,” “ulnar head,” “rheumatoid wrist,” “Darrach,” “ulnar stump,” and “pseudarthrosis.”

Studies were eligible for inclusion if they (i) were peer-reviewed primary reports (clinical case series or comparative studies) or evidence syntheses; (ii) described the Sauvé–Kapandji procedure or one of its bony or soft-tissue modifications, including its indications, technique, or outcomes; and (iii) were available in English and verifiable by a DOI or PMID. Foundational older sources, such as the original 1936 description and Kapandji’s technique papers, were retained where they remained the primary reference for a given point. Reports were excluded if they (i) did not specifically address the SK procedure; (ii) were available only as conference abstracts, editorials, or letters without full text; or (iii) contained claims that could not be confirmed against the corresponding full text. Titles and abstracts were initially screened for relevance, followed by full-text assessment of potentially eligible articles according to these criteria. Because this review was conducted as a narrative review rather than a systematic review, a formal PRISMA flow diagram was not prepared. The final synthesis included 32 references, of which 19 are primary clinical studies summarized in Table 1. The predominance of small, retrospective (Level IV) series, and the fact that study selection was performed by a single author without independent review, are acknowledged as limitations (Section 4).

## 3. The Sauvé–Kapandji Procedure: Evolution, Technique, Indications, and Outcomes

### 3.1. Historical Evolution

Historically, the most classic procedure for DRUJ reconstruction is distal ulnar resection (the Darrach procedure), first devised in 1912 for post-traumatic deformity [1,2]. By completely removing the ulnar buttress, however, it produced serious complications, such as ulnar carpal translation, radioulnar convergence, and ulnar stump instability, and proved limited in young or active patients [4,5]. To overcome these mechanical drawbacks, in 1936 Sauvé and Kapandji introduced the SK procedure, combining DRUJ arthrodesis with a proximal ulnar pseudarthrosis [3,27]. Initially confined to the treatment of recurrent dislocation, it became widely adopted for reconstruction of the rheumatoid wrist [4] and of osteoarthritis [13] as its biomechanical advantage—preserving ulnar bony support to prevent carpal ulnar translation and maintain physiological load transfer—became apparent.

As its clinical use expanded, the original SK procedure also faced new problems: proximal stump instability and, in RA patients, nonunion and inadequate bony support owing to severe osteopenia [17]. To address these, diverse modifications spanning both bone and soft tissue emerged over the following decades—one branch handling the distal ulna to reinforce the radiocarpal buttress, and another comprising various techniques to stabilize the proximal ulnar stump. This lineage continues to the present day [26], and the next section reorganizes these modifications within a two-axis framework.

### 3.2. The Two-Axis Framework

The accumulated modifications can be organized along two principal axes: handling of the distal ulna (bony axis) and stabilization of the proximal ulnar stump (soft-tissue axis) (Table 1). Although the framework separates these two dimensions for conceptual clarity, they should not be interpreted as biologically or surgically independent. Decisions made along the bony axis, particularly regarding the amount and position of ulnar resection, directly influence proximal ulnar stump stability and therefore affect considerations along the soft-tissue axis. This interaction is discussed further in Section 3.2.2 and Section 3.4.

#### 3.2.1. Distal Ulna Handling (Bony Axis)

In the traditional SK procedure, the articular cartilage of the DRUJ is removed, the ulnar head is fused directly to the radius, and a segment of the proximal ulnar metaphysis is resected to create a pseudarthrosis [4,8]. How much of the ulnar head to preserve (osteotomy level) and how much bone to resect (gap width) have evolved over time.

Reossification (bony bridging) of the pseudarthrosis is a hallmark complication of the SK procedure [2] because re-establishing bony continuity across the intended pseudarthrosis again restricts forearm rotation and defeats the purpose of the operation. In the early original technique, a relatively wide resection of about 3 cm was recommended to prevent reossification [27,28], but this caused serious stump instability. Kapandji himself therefore substantially reduced the resection width to about 7–20 mm [28]. Subsequent authors proposed more specific values; Kobayashi et al. recommended an osteotomy about 1.5 cm proximal to the ulnar tip with resection of about 1 cm [5]. Daecke et al. demonstrated that greater resection increases stump instability and recommended performing the osteotomy just proximal to the ulnar head and keeping the pseudarthrosis gap to about 10 mm [18]. Although no single absolute standard applies to every patient, recent clinical studies together suggest that preserving about 1–1.5 cm of the distal ulnar head for DRUJ arthrodesis and resecting the ulnar shaft to leave a pseudarthrosis gap of about 8–10 mm is the most widely used contemporary practice [18,21,26]. To prevent reossification, extra-periosteal resection that removes the adjacent periosteum and interosseous membrane [28] and interposition of the pronator quadratus (PQ) into the gap (see Section 3.2.2) are also recommended. When reossification nonetheless occurs, revision osteotomy may be required; in one large cohort, 4 of 57 wrists (about 7%) underwent revision osteotomy for reossification [9].

**Figure 1:**
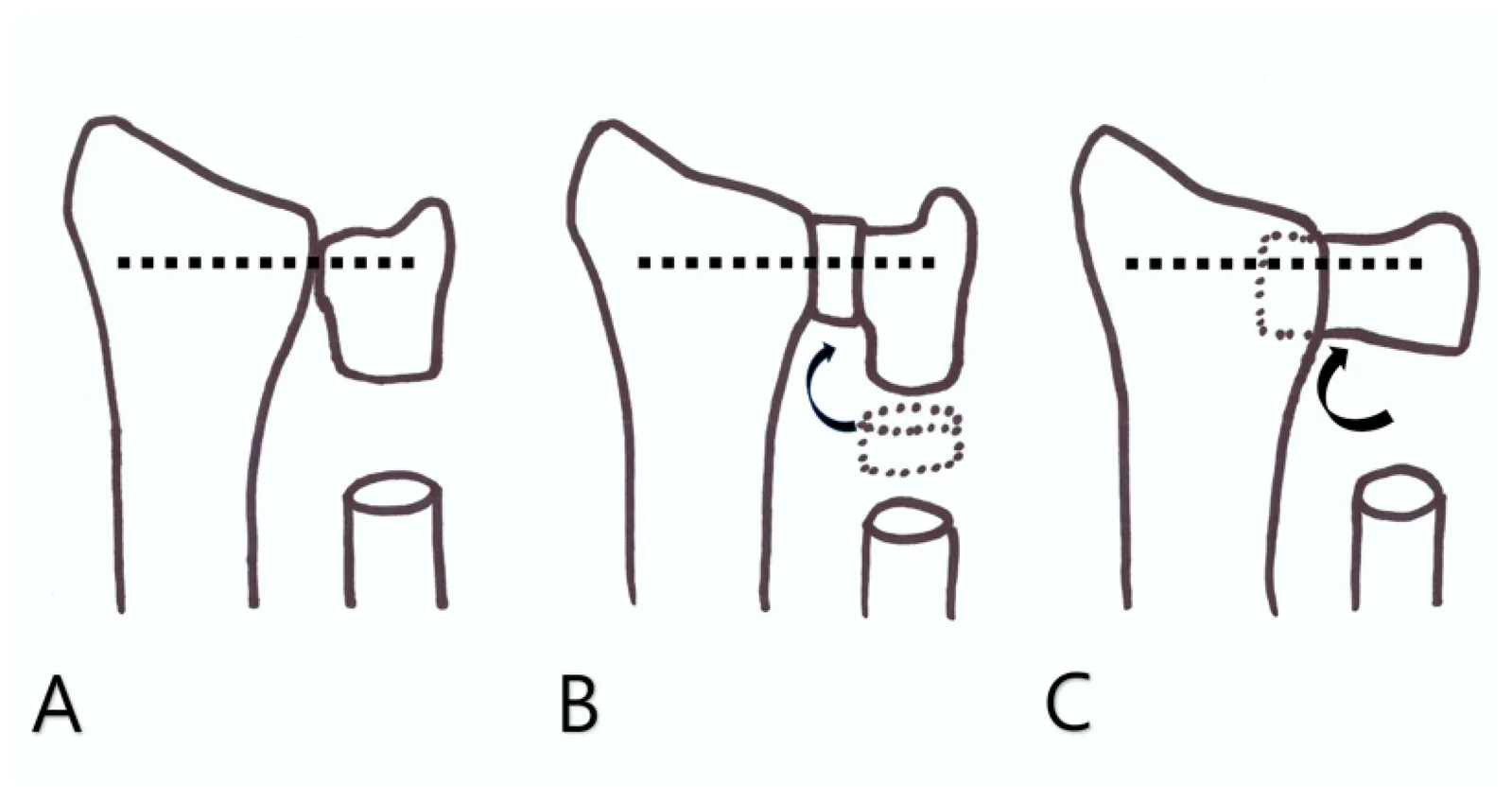
Schematic of the three bone-handling variants: (**A**) traditional Sauvé–Kapandji procedure, (**B**) fragment-interposition (Nakamura type), and (**C**) ulnar-head rotation (Fujita type). The dotted lines indicate the fixation screws.

In advanced RA, severe periarticular osteopenia and erosive loss of the DRUJ articular surfaces—involving the sigmoid notch of the radius as well as the ulnar head—leave poor-quality bone, so the remaining support is often inadequate to maintain a bony shelf for the carpus [17]. To restore the width of the radiocarpal buttress, two bony-structural modifications were devised, selected according to the severity of this erosion (Figure 1). For moderate deficiency, in which erosion of the sigmoid notch and/or the ulnar head narrows the shelf, the fragment-interposition type (Nakamura type; corresponding to modification-1 of Sakuma et al.) restores width by interposing part of the resected ulnar shaft between the sigmoid notch and the ulnar head [12,22]. For severe deficiency, in which the destruction of these articular surfaces leaves no usable shelf, the ulnar-head rotation type (Fujita type; modification-2 of Sakuma et al.) rotates the resected ulnar head by 90° and inserts it into a hole drilled in the distal radius to reconstruct a solid bony shelf [17,22]. Such structural reconstruction of the buttress is essential to restraining ulnar carpal translation and maintaining long-term joint alignment [17]. A wider shelf is not always better, however. Toyama et al. reported that an excessively wide shelf physically impedes dorsal repositioning of the extensor carpi ulnaris (ECU) tendon and may paradoxically cause ECU imbalance and palmar carpal subluxation, and on the basis of regression analysis recommended keeping the shelf width ratio (SWR) below 0.77 [20].

The materials for osteosynthesis have also advanced considerably. Early conventional metal screws frequently caused peri-screw bone resorption in poor-quality RA bone, requiring secondary screw removal in some cases [16]. Bioabsorbable poly-L-lactic acid (PLLA) screws were then introduced, achieving union without bone resorption and eliminating the need for hardware removal [16]; more recently the headless compression screw (HCS) has been preferred, eliminating screw-head prominence while providing rigid compression even in poor-quality bone without supplementary K-wire fixation [23].

#### 3.2.2. Proximal Stump Stabilization (Soft-Tissue Axis)

**Figure 2:**
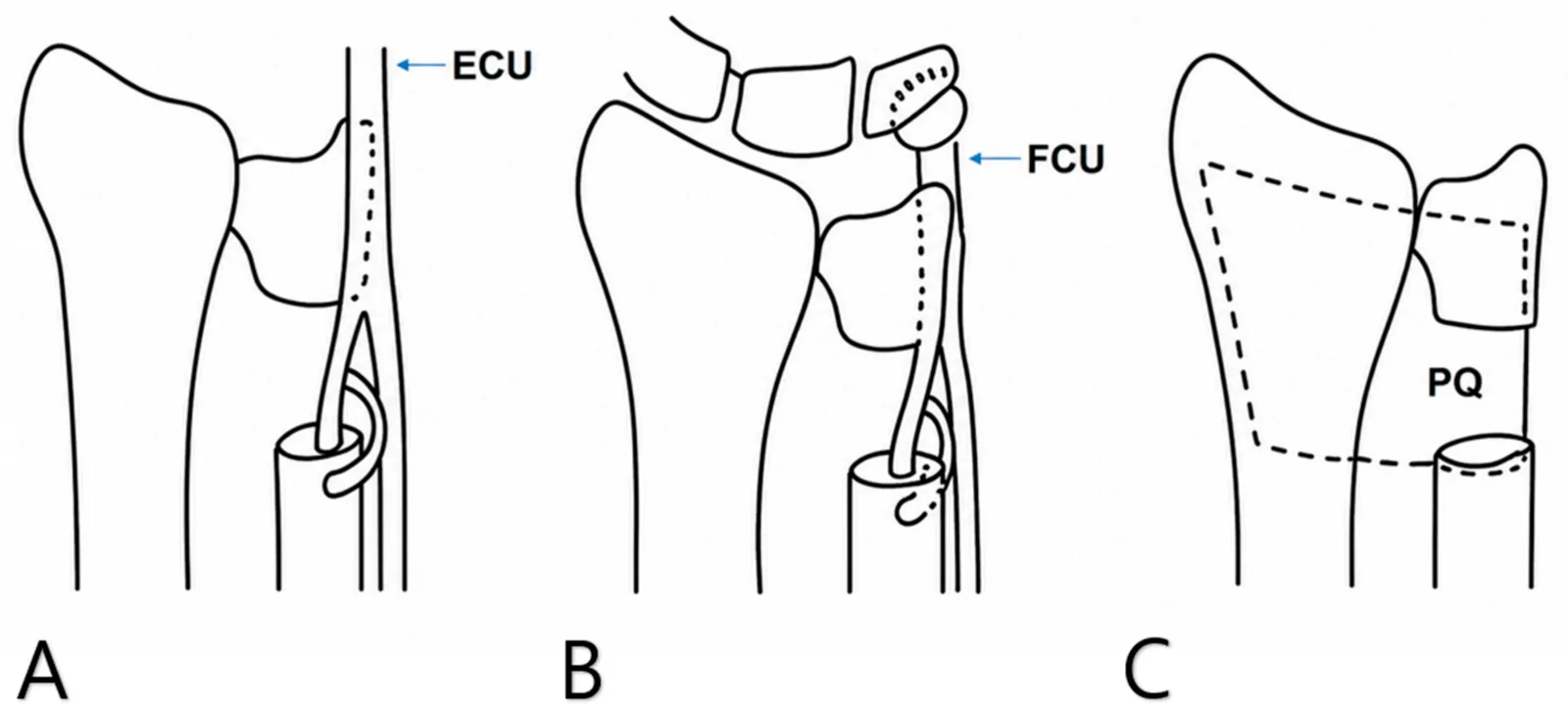
Representative soft-tissue stabilization techniques for the proximal ulnar stump after the Sauvé–Kapandji procedure. (**A**) Extensor carpi ulnaris (ECU) tenodesis: a split slip of the ECU tendon is passed through a drill hole in the proximal ulnar stump and fixed dorsally. (**B**) Flexor carpi ulnaris (FCU) tenodesis: a distally based split slip of the FCU tendon is secured around the proximal ulnar stump to improve stability. (**C**) Pronator quadratus (PQ) transfer: the PQ is transferred to provide soft-tissue coverage and stabilization of the proximal ulnar stump. Anteroposterior schematic, not to scale. The drawings are simplified conceptual illustrations and are not intended to depict all technical variations.

Ulnar impingement syndrome is a major complication that can arise after any procedure that resects the ulna. As a result of radioulnar convergence—in which the destabilized proximal ulnar stump drifts toward the radius—the stump impinges directly on the radial cortex, causing pain and grip weakness during forearm rotation [19]. Historically it was reported as a hallmark complication of the Darrach procedure, which abolishes ulnar support by completely resecting the ulnar head; but even after the SK procedure, which preserves the bony shelf, the same impingement and local pain can occur if the proximal stump is not adequately stabilized [8,19]. To prevent this, various soft-tissue stabilization techniques have been devised [4,21]. The principal soft-tissue stabilization techniques are summarized schematically in Figure 2. The most basic is soft-tissue preservation, in which the resected stump is covered and closed with periosteum and surrounding capsule without additional tendon transfer [17,24]. For more active stabilization, tenodesis—splitting a slip of the ECU tendon longitudinally, passing it through a hole in the proximal stump, and fixing it dorsally—became widely adopted [15], and techniques using the flexor carpi ulnaris (FCU) [14] or the PQ [21] were also introduced. In particular, dorsal transfer of the PQ acts as a dynamic stabilizer that depresses the stump palmarward during pronation while also functioning as an interposition spacer that physically blocks bony bridging (reossification) by filling the pseudarthrosis gap [21].

ECU tenodesis is one of the most widely used techniques, but whether it is a mandatory standard for every patient is debated. Kawabata et al. compared ECU-stabilized and non-stabilized groups in RA patients and reported no significant difference in final clinical or radiographic outcomes apart from earlier pain relief in the stabilized group [19], and Ikeda et al. obtained a stable, painless stump using periosteal and soft-tissue coverage alone, omitting tenodesis [24]. Giberson-Chen et al. likewise found ECU tenodesis and PQ transfer similar in QuickDASH, range of motion, and complication rate, suggesting that ECU fixation is not particularly superior to other soft-tissue techniques [9].

This debate suggests that, more than the mode of soft-tissue reconstruction, the amount and position of ulnar resection itself (see Section 3.2.1) may be the more fundamental determinant of stump stability. Indeed, Daecke et al. showed that the amount of proximal ulnar shortening is a key predictor of instability and poor clinical outcome (each 1 mm of resection raising the risk of pain by about 10%) and recommended limiting this shortening, measured from the wrist joint line, to about 35 mm [18]. Subsequent clinical studies have actively adopted this mechanical principle: Kawabata et al., citing Daecke’s criterion, considered that osteotomy 30 mm from the joint line may have contributed to stump stability [19], and Papp et al. likewise emphasized that performing the ulnar resection conservatively, distal to the PQ insertion, lowers the risk of instability [8].

### 3.3. Outcomes and Comparative Evidence

The SK procedure is a salvage option that markedly relieves pain and improves forearm rotation across DRUJ disorders of diverse etiology, and recent series continue to confirm its utility [9,29]. Postoperative pronation and supination improve appreciably, whereas wrist flexion consistently tends to decrease [24,26]. In patients without tendon rupture, Kato et al. showed that because the DRUJ itself contributes biomechanically to wrist flexion–extension, fusing it inevitably limits flexion (49°→40°), whereas the Darrach procedure, which abolishes radioulnar impingement by resection, may instead increase flexion and extension [25].

Direct comparison of SK and Darrach informs clinical choice. Although SK is theoretically expected to better restrain ulnar carpal translation and preserve grip by maintaining the ulnar buttress, a long-term radiographic comparison in RA patients found no significant difference between the procedures in the progression of carpal collapse (carpal height) or ulnar translation—neither halts the natural course of RA [5]. Recent systematic review and meta-analysis likewise found the two procedures broadly equivalent in major outcomes including QuickDASH, grip strength, range of motion, and satisfaction [10,11]. Optimization of radiographic outcomes depends on the geometry of the fixed ulnar head and on stump management (see Section 3.2); imparting radial inclination to the fixed ulnar head with adequate—but not excessive—support width is associated with less postoperative carpal translation [20,22]. Even so, by preserving a wider radiocarpal bony shelf, the SK procedure can mechanically resist frank ulnocarpal dislocation and may therefore be preferred in patients with marked preoperative ulnar translation or high functional demand [5].

These advantages are offset by complications. Long-term series report substantial revision rates driven by proximal ulnar stump instability—up to 18% in one comparative cohort and 6 of 15 wrists in a 13-year series—leading some authors to restrict the SK procedure to selected cases [6,7]. Advances in fixation and pharmacotherapy may mitigate some of these problems. The HCS achieves close to 100% union without supplementary K-wire fixation and without screw-head complications even in osteopenic rheumatoid wrists [23]. Moreover, multivariate analysis by Okabe et al. showed that perioperative biologic agents (TNF inhibitors) independently shorten the time to bone union, whereas high-dose methotrexate and NSAIDs delay it—indicating that strategic perioperative medication management, not surgical technique alone, is important to a successful SK procedure [30]. The major complications of the procedure, together with their reported incidence, risk factors, preventive strategies, and management, are summarized in Table 2.

### 3.4. An Indication-Based Decision Framework and Research Agenda

**Figure 3:**
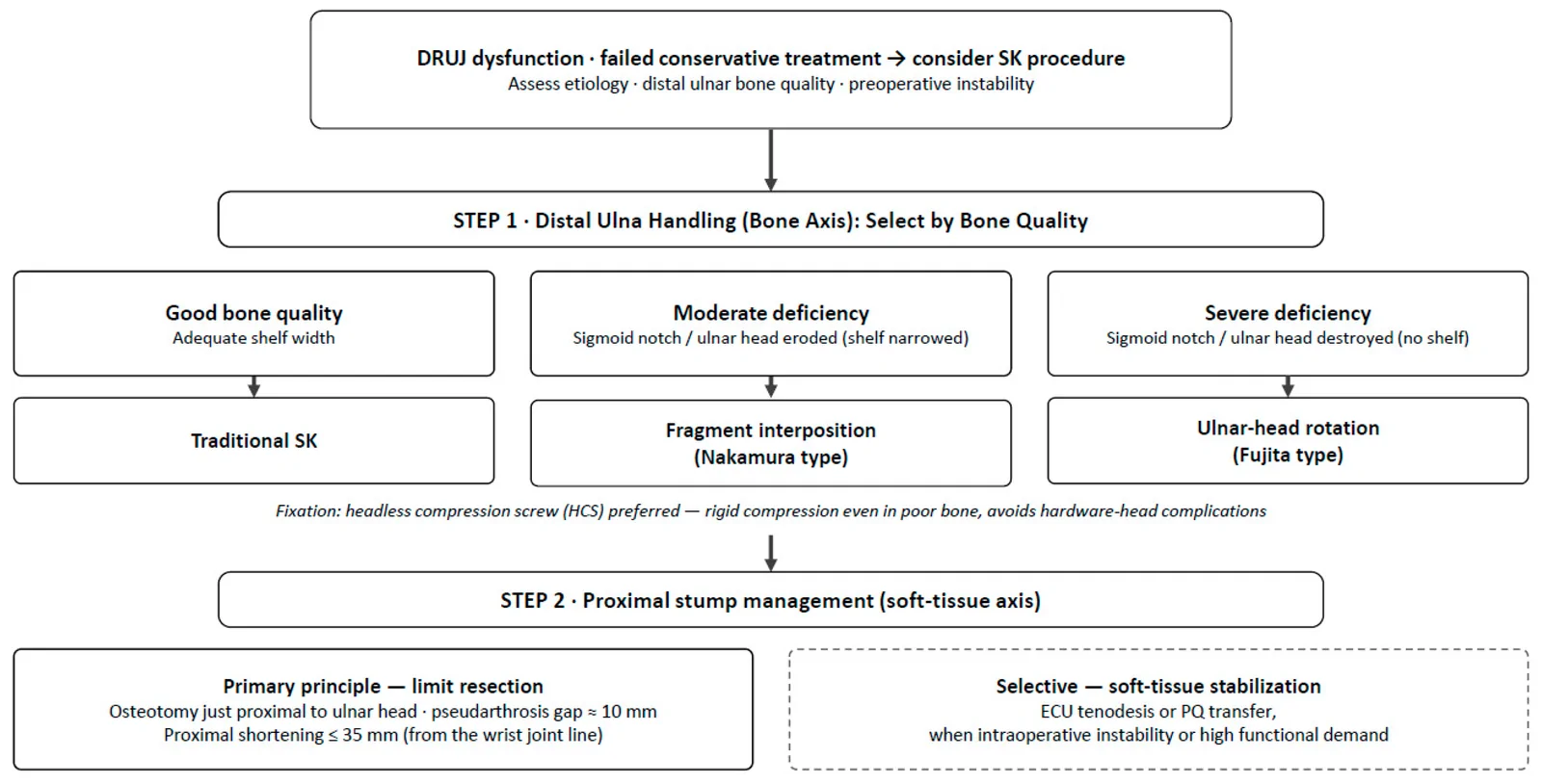
Indication-based decision framework (flowchart)—stepwise selection by etiology, bone quality, and preoperative instability. Bone quality is categorized according to the degree of distal ulnar and sigmoid notch preservation on preoperative radiographs, supplemented by CT and confirmed intraoperatively where necessary; in rheumatoid arthritis, the Larsen grading system may serve as a practical radiographic reference (good, approximately Larsen 0–III; moderate, approximately IV; severe, approximately V), although these categories have not been formally validated for selecting among the SK modifications.

Previous higher-level syntheses of these procedures have compared the SK and Darrach procedures in terms of pooled clinical outcomes but have treated the SK procedure as a single entity; they did not distinguish among its bony and soft-tissue modifications, define their differing indications, or offer guidance on selecting among them [10,11]. Earlier narrative overviews, in turn, have largely organized the modifications chronologically or by individual technique. The novelty of the present review lies not in proposing another surgical technique but in providing a clinically oriented conceptual framework that integrates the existing modifications according to their indications and biomechanical rationale. The bony-structural modifications discussed here (Nakamura and Fujita types) were developed mainly to compensate for the osteopenic, erosive bone loss of RA. Although the SK procedure and its modifications developed largely within the context of rheumatoid disease, contemporary indications increasingly include non-rheumatoid conditions in which bone stock is typically preserved. In post-traumatic DRUJ arthritis and chronic post-traumatic DRUJ instability, the SK procedure has been reported to relieve ulnar-sided pain and restore forearm rotation while preserving ulnar support, and it was for chronic instability that the procedure was originally described [12,27]. Favorable results have been reported in chronic post-traumatic derangement [14], and comparative and case–control studies confirm reliable outcomes in post-traumatic DRUJ dysfunction, including direct comparison with the Darrach procedure [31] and comparison of different methods of proximal ulnar stump stabilization [32]. In distal radius malunion with positive ulnar variance, the technique can be combined with leveling of the ulnar head, as in Kapandji’s original description for post-Colles derangement [28]. Because the distal ulnar bone is generally intact in these settings, the traditional SK may be sufficient in many patients without bony augmentation; the stepwise bony-axis selection is therefore most relevant to rheumatoid wrists, whereas the principles of limited resection and proximal stump stabilization apply across all etiologies. Synthesizing these points, selection of an SK modification is determined by the combination of etiology, the bone quality of the DRUJ articular surfaces (the sigmoid notch and ulnar head; good–moderate–severe deficiency), and the presence of preoperative instability, and can be reduced to two sequential decisions (Figure 3). First, distal bone handling is determined by DRUJ bone erosion—traditional SK for good bone with adequate shelf width; the fragment-interposition (Nakamura) type for moderate deficiency from sigmoid-notch and/or ulnar-head erosion; and the ulnar-head rotation (Fujita) type for severe deficiency, in which marked destruction of these articular surfaces leaves no usable shelf. For fixation, the HCS is preferred as it provides rigid compression with a high union rate even in poor-quality RA bone while avoiding screw-head complications [22,23]. Second, proximal stump management takes limitation of resection as the primary preventive measure—performing the distal osteotomy just proximal to the ulnar head and keeping the pseudarthrosis gap at about 10 mm with proximal shortening within about 35 mm of the wrist joint line [18]. Soft-tissue stabilization (ECU tenodesis or PQ transfer) may be added selectively when intraoperative instability is demonstrated or functional demand is high, although its necessity remains debated [19].

For practical application, preoperative assessment of bone quality may be based primarily on conventional radiographs, with computed tomography used when bone stock cannot be adequately assessed on plain films and with confirmation at operation. Good bone quality refers to preservation of the sigmoid notch and ulnar head with sufficient bone stock for conventional fixation; moderate deficiency refers to advanced erosive change that narrows the bony shelf while leaving some bony support; and severe deficiency refers to end-stage destruction with loss of the bony shelf. In rheumatoid arthritis, the degree of bone deficiency may be estimated using the Larsen grading system as a practical radiographic reference: good bone quality generally corresponds to lower Larsen grades (approximately 0–III) with preserved bone stock, moderate deficiency to advanced erosive change (approximately Larsen IV), and severe deficiency to end-stage destruction (approximately Larsen V). Because the Larsen grading system, which has been widely used to classify the severity of rheumatoid joint destruction and has been applied in studies of advanced rheumatoid wrists [26], has not been validated specifically for selecting among the SK modifications, these categories are presented as a pragmatic clinical framework rather than a validated grading system and require prospective validation.

It should be emphasized that these numerical values, namely a shelf width ratio below 0.77 [20], a pseudarthrosis gap of approximately 10 mm, and proximal shortening within approximately 35 mm [18], are each derived from single, small, retrospective studies and have not been prospectively validated or compared across techniques. They should therefore be regarded as provisional reference values rather than validated clinical thresholds, and they require confirmation in future comparative studies. Likewise, the two-axis framework itself and the mapping of bone-quality categories to specific modifications represent the authors’ organizational synthesis, intended to structure the existing literature and to generate testable hypotheses rather than to provide validated clinical recommendations or practice guidelines. The framework is thus intended as an organizational and decision-support model rather than a representation of two fully independent biological processes. Accordingly, the proposed framework should be regarded as an aid to clinical reasoning and hypothesis generation rather than as a prescriptive treatment algorithm.

Each branch of this framework maps directly onto an as-yet-unmet research question. (1) No study has directly compared the bony-handling variants stratified by bone quality (e.g., Larsen grade or shelf width); given that existing syntheses compare only SK with Darrach, this is the core gap [10,11]. (2) The necessity of stump stabilization must be resolved by randomized comparison using a standardized instability measure. (3) The resection thresholds (35 mm of shortening, 10 mm gap) derive from a single retrospective study and require prospective validation [18]. Given that the current evidence is predominantly Level IV, this two-axis framework is offered not as an established guideline but as a hypothesis-generating conceptual framework awaiting validation. Accordingly, it should be regarded as an aid to clinical reasoning and hypothesis generation rather than as a prescriptive treatment algorithm.

## 4. Limitations

This review has several limitations, both in its methodology and in the underlying literature. First, as a narrative rather than a systematic review, it did not follow a pre-registered search and selection protocol, and study selection was performed by a single author without independent review, which may have introduced selection bias. In addition, no formal quality or risk-of-bias appraisal of the included studies was undertaken; consistent with a narrative approach, studies were synthesized qualitatively rather than graded with structured instruments such as MINORS or ROBINS-I, and the reliability of individual reports could therefore not be systematically assessed. 

Second, the available evidence consists predominantly of small, single-center, retrospective (Level IV) case series, often without control groups and with heterogeneity in underlying disease (RA, osteoarthritis, and trauma), technical variant, fixation method, outcome measures, and follow-up duration, which precludes quantitative pooling or direct comparison. Third, no study has compared the modifications head-to-head under equivalent conditions, and a clinical framework constructed from such lower-level evidence carries an inherent risk of overgeneralization; the proposed two-axis framework is therefore a conceptual, hypothesis-generating proposal that structures existing evidence rather than a construct validated by comparative data. Fourth, some quantitative values cited (e.g., a shelf width ratio of 0.77; proximal shortening of 35 mm and a pseudarthrosis gap of 10 mm) derive from individual single studies and have not been prospectively validated. 

In addition, a substantial proportion of the primary studies informing this review, particularly those describing the bony modifications of the Sauvé–Kapandji procedure, originated from Japanese and, more broadly, East Asian centers. This geographic concentration may reflect regional patterns of clinical practice and reporting and may limit the generalizability of the proposed framework to other populations, healthcare systems, and surgical settings. For these reasons, the proposed framework should be interpreted as a hypothesis-generating tool intended to organize existing observations and to guide future research rather than as a validated clinical decision rule, and prospective comparative studies will be required before it can be recommended for routine surgical decision-making. Accordingly, the conclusions of the present review should be interpreted in the context of these methodological limitations.

## 5. Conclusions

The SK procedure is a reliable salvage option for DRUJ disorders that relieves pain and restores forearm rotation while preserving ulnar bony support. This review integrated the scattered modifications along two axes—distal bone handling and proximal soft-tissue stabilization—and linked them to patient etiology and bone quality in an indication-based decision framework. Clinically, a reasonable approach is to select stepwise from the traditional SK to the fragment-interposition and ulnar-head rotation types according to bone quality, to take avoidance of excessive resection as the primary principle for the proximal stump, and to apply soft-tissue stabilization selectively. Current systematic reviews and meta-analyses report broadly equivalent major outcomes for the SK and Darrach procedures; however, these pooled analyses combine heterogeneous populations, and this overall equivalence should not be assumed to apply uniformly to every subgroup. In particular, in rheumatoid patients at high risk of ulnar carpal translation, the SK procedure, by preserving ulnar bony support, may offer theoretical biomechanical advantages and has been associated with favorable outcomes in some clinical series, so the two procedures may not be equivalent in this specific population. Treatment decisions should therefore integrate the available evidence with patient-specific pathology and surgical objectives rather than relying solely on pooled comparisons. However, because direct comparative evidence among the modifications is lacking and the current level of evidence is low, this framework is offered as a testable, hypothesis-generating framework rather than an established guideline. Prospective comparative studies stratified by bone quality, randomized trials on the necessity of stump stabilization, and validation of resection thresholds and perioperative medication management remain priorities for future research.

## Figures and Tables

**Table 1:** Two-axis classification of representative studies on the Sauvé–Kapandji procedure.

Study (Year) [Ref.]	Design *n*	Etiology	Bone Handling (Axis 1)	Stump Stabilization (Axis 2)	Fixation	Key Finding
Nakamura 1992 [12]	Retro 15	Chronic dislocation	Fragment interposition	Periosteum	Metal screw	RU convergence/stump instability in all; pain in 4/15
Vincent 1993 [4]	Retro 17 (21 wrists)	RA	Traditional	Soft tissue	Metal screw	Prevented ulnar translation, improved rotation
Minami 1995 [13]	Retro 15 patients	Primary OA	Traditional	—	Metal screw	Good pain/function in OA
Lamey & Fernandez 1998 [14]	Retro 18 patients	Post-traumatic	Traditional (modified)	FCU	Metal screw	Good results in post-traumatic DRUJ
Minami 2000 [15]	Retro 13 wrists	RA/OA	Traditional	ECU tenodesis	Metal screw	Introduced ECU tenodesis modification
Nakamura K 2002 [16]	Retro 43 (23 PLLA/20 Metal)	RA	Traditional	—	PLLA absorbable screw	No peri-screw resorption; no removal needed
Fujita 2005 [17]	Retro 56 (66 wrists)	RA	Ulnar-head rotation	—	Metal screw	Reconstructed bony shelf in bone deficiency
Kobayashi 2005 [5]	Retro comparison (vs Darrach) 20 (26 wrists)	RA	Traditional	—	Metal screw	No difference in carpal collapse/ulnar translation
Daecke 2006 [18]	Retro 44	Mixed	Traditional	Various	Metal screw	More resection → more instability; shortening ≤35 mm
Kawabata 2010 [19]	Retro comparison (ECU ±) 41	RA	Ulnar-head rotation	ECU vs. none	Metal screw	No difference except early pain; 30 mm osteotomy
Toyama 2011 [20]	Retro 20 (24 wrists)	RA	Ulnar-head rotation	—	Metal screw	Recommended SWR < 0.77
Papp 2013 [8]	Retro long-term 14	RA	Traditional	ECU + PQ interposition	Metal screw	Good long-term; no translation/subluxation
Uerpairojkit 2014 [21]	Retro 10	RA	Traditional	PQ transfer	1 cancellous screw	Sagittal stable; coronal convergence on loading (asymptomatic)
Sakuma 2016 [22]	Retro 40 patients	RA	Traditional/interposition/rotation	—	Metal screw	Fixed ulnar-head position associated with carpal translation
Maeda 2018 [23]	Retro 41	RA	Fragment interposition	PQ + periosteum	HCS	High union rate; no hardware complications
Ikeda 2018 [24]	Retro 28 (32 wrists)	RA/OA	Traditional	Periosteal coverage	1 HCS	Stable, painless stump without tenodesis
Giberson-Chen 2020 [9]	Retro comparison (ECU vs. PQ) 57	Mixed	Traditional	ECU vs. PQ	2 HCS	Two groups similar; 7% reosteotomy for reossification
Kato 2021 [25]	Retro comparison (vs. Darrach) 46	Mixed	Traditional	—	Metal screw or HCS	Flexion 49°→40°; Darrach increases flexion/extension
Liu 2026 [26]	Retro 6 (9 wrists)	Advanced RA	Traditional (no graft)	ECU only	2 HCS	Radiographic convergence/volar migration progress but function improves

Axis 1 (bony) = distal ulna handling: traditional/fragment-interposition (Nakamura type)/ulnar-head rotation (Fujita type). Axis 2 (soft tissue) = proximal stump stabilization: periosteal preservation/ECU/FCU/PQ/combination. “—” indicates that the item was not specified or was not a focus of the study. Retro, retrospective. *n* denotes the number of wrists unless otherwise stated; where both patients and wrists are reported, *n* refers to the number of patients, with the number of wrists in parentheses.

**Table 2:** Major complications of the Sauvé–Kapandji procedure, associated risk factors, preventive strategies, and management.

Complication	Reported Incidence	Major Risk Factors	Preventive Strategies	Management
Proximal ulnar stump instability/ulnar impingement	Revision for instability in up to ~18% in one comparative cohort [6] and in 6 of 15 wrists in a 13-year series [7]; ulnar stump pain in 4/15 (26.7%) [12]; radioulnar convergence radiographically common [7,12,21]	Excessive or too-proximal resection (large shortening); inadequate stump stabilization	Limit resection: osteotomy just proximal to the ulnar head; pseudarthrosis gap ~10 mm and proximal shortening ≤35 mm (provisional reference values *) [18]; selective ECU [15], FCU [14], or PQ [21] stabilization	Secondary ECU/FCU tenodesis or PQ transfer; revision
Reossification (bony bridging) of the pseudarthrosis	Revision osteotomy in 7.0% (4/57) [9]	Too-small pseudarthrosis gap; incomplete removal of periosteum/interosseous membrane	Adequate pseudarthrosis gap (~10 mm *) [18]; extra-periosteal resection removing periosteum and interosseous membrane [28]; PQ interposition [21]	Revision osteotomy [9]
Nonunion/delayed union of the DRUJ arthrodesis	Nonunion in 6 of 19 wrists (31.6%) with conventional screws in an earlier series [17]; high union rates reported with headless compression screws [23], and no nonunions in a contemporary cohort [9]	Poor (osteopenic) RA bone; high-dose methotrexate or NSAIDs [30]	Rigid compression fixation (HCS) [23] with cancellous bone grafting; perioperative optimization (biologic/TNF-inhibitor therapy accelerates union, whereas high-dose MTX and NSAIDs delay it) [30]	Revision arthrodesis with autologous bone grafting
Extensor tendon attrition/rupture	Reported (tendon impingement over a prominent or unstable stump) [8,19]	Prominent/unstable proximal stump; osteotomy performed too distally	Osteotomy ~25–30 mm proximal to the joint line; periosteal and soft-tissue coverage of the stump [19]	Tendon transfer (e.g., extensor indicis proprius) or free tendon graft
Hardware-related (peri-screw resorption, screw-head irritation)	Peri-screw bone resorption in 5/20 (25%) with metal screws [16]; screw-head discomfort in 2/21 (9.5%) with cannulated cancellous screws [23]; none with PLLA [16] or HCS [23]	Osteopenic RA bone; prominent screw heads	Bioabsorbable (PLLA) screws [16] or headless compression screws [23]	Hardware removal after union if symptomatic

* Values are derived from small retrospective studies and should be regarded as provisional reference values rather than validated thresholds. The reported frequencies and numerical values are derived predominantly from individual Level IV studies and should be interpreted as representative observations rather than validated estimates.

## Data Availability

No new data were created or analyzed in this study. Data sharing is not applicable to this article.
